# Supplementary material for: Pyruvate Dehydrogenase Kinase 1 inhibition mediated oxidative phosphorylation enhancement in cartilage promotes osteoarthritis progression
Source: BMC Musculoskelet Disord. 2023 Jul 20;24:597. doi: 10.1186/s12891-023-06585-6 (PMC10357736; doi:10.1186/s12891-023-06585-6)
Supplement: Supplementary file 1 — Additional file 1. [file 12891_2023_6585_MOESM1_ESM.zip › KEGG.Gsea.1653622432464/KEGG_TRYPTOPHAN_METABOLISM.html]

Details for gene set KEGG\_TRYPTOPHAN\_METABOLISM[GSEA]

|  || Dataset | 55457\_collapsed\_to\_symbols.cls.cls #OA\_versus\_normal.cls.cls #OA\_versus\_normal\_repos |
| Phenotype | cls.cls#OA\_versus\_normal\_repos |
| Upregulated in class | OA |
| GeneSet | KEGG\_TRYPTOPHAN\_METABOLISM |
| Enrichment Score (ES) | 0.38332263 |
| Normalized Enrichment Score (NES) | 1.4253742 |
| Nominal p-value | 0.061926607 |
| FDR q-value | 0.09772028 |
| FWER p-Value | 0.936 |
Table: GSEA Results Summary

  

Fig 1: Enrichment plot: KEGG\_TRYPTOPHAN\_METABOLISM      
 Profile of the Running ES Score & Positions of GeneSet Members on the Rank Ordered List

  

| SYMBOL | TITLE | RANK IN GENE LIST | RANK METRIC SCORE | RUNNING ES | CORE ENRICHMENT || 1 | KMO | kynurenine 3-monooxygenase [Source:HGNC Symbol;Acc:HGNC:6381] | 694 | 0.595 | 0.0134 | Yes |
| 2 | ALDH2 | aldehyde dehydrogenase 2 family member [Source:HGNC Symbol;Acc:HGNC:404] | 1003 | 0.517 | 0.0479 | Yes |
| 3 | ACAT1 | acetyl-CoA acetyltransferase 1 [Source:HGNC Symbol;Acc:HGNC:93] | 1017 | 0.513 | 0.1050 | Yes |
| 4 | ALDH3A2 | aldehyde dehydrogenase 3 family member A2 [Source:HGNC Symbol;Acc:HGNC:403] | 1263 | 0.465 | 0.1386 | Yes |
| 5 | CAT | catalase [Source:HGNC Symbol;Acc:HGNC:1516] | 1731 | 0.394 | 0.1468 | Yes |
| 6 | TDO2 | "tryptophan 2,3-dioxygenase [Source:HGNC Symbol;Acc:HGNC:11708]" | 1783 | 0.387 | 0.1866 | Yes |
| 7 | ECHS1 | "enoyl-CoA hydratase, short chain 1 [Source:HGNC Symbol;Acc:HGNC:3151]" | 1925 | 0.369 | 0.2175 | Yes |
| 8 | AOC1 | amine oxidase copper containing 1 [Source:HGNC Symbol;Acc:HGNC:80] | 1992 | 0.362 | 0.2533 | Yes |
| 9 | ALDH7A1 | aldehyde dehydrogenase 7 family member A1 [Source:HGNC Symbol;Acc:HGNC:877] | 2158 | 0.343 | 0.2792 | Yes |
| 10 | MAOB | monoamine oxidase B [Source:HGNC Symbol;Acc:HGNC:6834] | 2192 | 0.339 | 0.3151 | Yes |
| 11 | KYNU | kynureninase [Source:HGNC Symbol;Acc:HGNC:6469] | 2502 | 0.303 | 0.3253 | Yes |
| 12 | ALDH9A1 | aldehyde dehydrogenase 9 family member A1 [Source:HGNC Symbol;Acc:HGNC:412] | 2795 | 0.270 | 0.3332 | Yes |
| 13 | WARS2 | "tryptophanyl tRNA synthetase 2, mitochondrial [Source:HGNC Symbol;Acc:HGNC:12730]" | 3014 | 0.248 | 0.3443 | Yes |
| 14 | AANAT | aralkylamine N-acetyltransferase [Source:HGNC Symbol;Acc:HGNC:19] | 3017 | 0.248 | 0.3722 | Yes |
| 15 | HAAO | "3-hydroxyanthranilate 3,4-dioxygenase [Source:HGNC Symbol;Acc:HGNC:4796]" | 3209 | 0.229 | 0.3833 | Yes |
| 16 | HADHA | hydroxyacyl-CoA dehydrogenase trifunctional multienzyme complex subunit alpha [Source:HGNC Symbol;Acc:HGNC:4801] | 4163 | 0.141 | 0.3252 | No |
| 17 | HADH | hydroxyacyl-CoA dehydrogenase [Source:HGNC Symbol;Acc:HGNC:4799] | 5088 | 0.065 | 0.2608 | No |
| 18 | CYP1B1 | cytochrome P450 family 1 subfamily B member 1 [Source:HGNC Symbol;Acc:HGNC:2597] | 5925 | 0.000 | 0.1959 | No |
| 19 | ASMT | acetylserotonin O-methyltransferase [Source:HGNC Symbol;Acc:HGNC:750] | 5986 | -0.003 | 0.1916 | No |
| 20 | AOX1 | aldehyde oxidase 1 [Source:HGNC Symbol;Acc:HGNC:553] | 6243 | -0.024 | 0.1744 | No |
| 21 | ACAT2 | acetyl-CoA acetyltransferase 2 [Source:HGNC Symbol;Acc:HGNC:94] | 6291 | -0.027 | 0.1737 | No |
| 22 | ALDH1B1 | aldehyde dehydrogenase 1 family member B1 [Source:HGNC Symbol;Acc:HGNC:407] | 6551 | -0.046 | 0.1588 | No |
| 23 | IDO1 | "indoleamine 2,3-dioxygenase 1 [Source:HGNC Symbol;Acc:HGNC:6059]" | 7782 | -0.140 | 0.0790 | No |
| 24 | EHHADH | enoyl-CoA hydratase and 3-hydroxyacyl CoA dehydrogenase [Source:HGNC Symbol;Acc:HGNC:3247] | 7968 | -0.153 | 0.0819 | No |
| 25 | GCDH | glutaryl-CoA dehydrogenase [Source:HGNC Symbol;Acc:HGNC:4189] | 8180 | -0.170 | 0.0847 | No |
| 26 | CYP1A1 | cytochrome P450 family 1 subfamily A member 1 [Source:HGNC Symbol;Acc:HGNC:2595] | 8195 | -0.171 | 0.1029 | No |
| 27 | MAOA | monoamine oxidase A [Source:HGNC Symbol;Acc:HGNC:6833] | 8271 | -0.177 | 0.1171 | No |
| 28 | OGDH | oxoglutarate dehydrogenase [Source:HGNC Symbol;Acc:HGNC:8124] | 8503 | -0.194 | 0.1211 | No |
| 29 | DDC | dopa decarboxylase [Source:HGNC Symbol;Acc:HGNC:2719] | 8605 | -0.202 | 0.1361 | No |
| 30 | WARS1 | tryptophanyl-tRNA synthetase 1 [Source:HGNC Symbol;Acc:HGNC:12729] | 10289 | -0.350 | 0.0448 | No |
| 31 | OGDHL | oxoglutarate dehydrogenase L [Source:HGNC Symbol;Acc:HGNC:25590] | 10754 | -0.399 | 0.0539 | No |
| 32 | TPH1 | tryptophan hydroxylase 1 [Source:HGNC Symbol;Acc:HGNC:12008] | 11223 | -0.460 | 0.0696 | No |
| 33 | CYP1A2 | cytochrome P450 family 1 subfamily A member 2 [Source:HGNC Symbol;Acc:HGNC:2596] | 11719 | -0.536 | 0.0918 | No |
Table: GSEA details [plain text format]

  

Fig 2: KEGG\_TRYPTOPHAN\_METABOLISM      
 Blue-Pink O' Gram in the Space of the Analyzed GeneSet

  

Fig 3: KEGG\_TRYPTOPHAN\_METABOLISM: Random ES distribution      
 Gene set null distribution of ES for **KEGG\_TRYPTOPHAN\_METABOLISM**
